# The Impact of the COVID-19 Self-Isolation Policy on the Occupations of Vulnerable Groups

**DOI:** 10.3390/ijerph18126452

**Published:** 2021-06-15

**Authors:** Amirudin Amirudin, Mariusz Urbański, Jumadil Saputra, Muhamad Deni Johansyah, Latip Latip, Ahmad Tarmizi, Teuku Afrizal

**Affiliations:** 1 Faculty of Humanities, Tembalang, Universitas Diponegoro, Semarang 50275, Indonesia; amirudin@lecturer.undip.ac.id; 2 Faculty of Civil Engineering, Częstochowa University of Technology, Akademicka 3, PL 42-200 Czestochowa, Poland; murbanski@interia.eu; 3 Faculty of Business, Economics and Social Development, Universiti Malaysia Terengganu, Kuala Nerus, Terengganu 21030, Malaysia; 4 Faculty of Mathematics and Natural Sciences, Universitas Padjadjaran, Jatinangor, Sumedang, West Java 45363, Indonesia; muhamad.deni@unpad.ac.id; 5 Sekolah Tinggi Ilmu Administrasi Publik, Dumai Selatan, Dumai, Riau 28826, Indonesia; latip.stiadmi@gmail.com; 6 Faculty of Social and Political Sciences, Universitas Islam Riau, Pekanbaru, Riau 28284, Indonesia; tarmiziuir@soc.uir.ac.id; 7 Faculty of Social and Political Sciences, Universitas Diponegoro, Semarang, Jawa Tengah 50275, Indonesia

**Keywords:** coronavirus disease 2019 (COVID-19) pandemic, self-isolation policy, rational and non-rational thinking model, occupational, vulnerable groups

## Abstract

Today, the spread of the Coronavirus 2019 (COVID-19) pandemic continues to impact on world public health and bring about considerable human suffering partly due to government policies on reducing the spread. COVID-19 has significantly affected human health and it has impacted on the occupation of vulnerable groups such as tour guides, drivers and shop assistants. Of these, the present study aims to investigate the impact of the COVID-19 self-isolation policy on the occupation of vulnerable groups in Semarang City, Indonesia. To achieve this objective, this study uses a qualitative method with an ethnography approach considering a rational or non-rational thinking model. The binary opposition thinking pattern pioneered by Lévi-Strauss was used in the interview process with 25 informants in Semarang City, Indonesia. The data analyzed the response pattern of informants through the taxonomy analysis. Three levels of vulnerability among groups relating to occupation were identified; jobs lost, income decreased, and delayed salary. The result of the analysis found that the group who obeyed self-isolation was categorized as a rational thinking; these groups stay at home, do not go to work, and have no income. Besides that, the group who ignored self-isolation is categorized as non-rational thinking; they work, as usual, get their salary, and believe that the COVID-19 pandemic is a disaster and they pray for their safety to God. In conclusion, COVID-19 brings a significant impact on occupation in the forms of postponing, declining, and missing income besides the health effects among vulnerable groups in Semarang city, Indonesia. In avoiding COVID-19 infection, the circumstances of vulnerable groups are worse when self-isolation is required. Thus, this study suggests that the government needs to assist vulnerable groups by focusing on strategic policies, such as strategies for survival, providing access to basic needs, including health, and offering livelihood plans by providing access to medical services and other source of income.

## 1. Introduction

The spread of the Coronavirus disease 2019 (COVID-19) pandemic continues to escalate throughout the world [1]. Officially, it emerged in China since the first human case was reported in Wuhan in December 2019 [2]. The COVID-19 pandemic is severely impacting on world public health and is causing considerable human suffering [3]. Hence, more than 200 countries and regions are affected by the epidemic, with the number of infections and deaths still increasing [4,5]. In April 2020, 213 countries and territories are affected worldwide, the number of positive cases has reached 13,378,853, and 580,045 patients have died. The country most infected by COVID-19 is the United States with 7,016,851 deaths, Europe has 2,987,256, Africa 523,403, and Southeast Asia 1,268,923 [2]. The number of cases of global deaths that have occurred is 6.3 per cent. 

As of 15 July 2020, Indonesia has reported that the number of positive COVID-19 patient cases continue to grow, and the number of people infected with COVID-19 has reached 81,667, with 40,345 patients recovered, and 3873 patients who have died. The current death rate is 4.74 per cent [6]. This leads Indonesia to have the highest COVID-19 cases in Southeast Asia, after the Philippines and Singapore, and rank six in Asia according to the death numbers. Indonesia has conducted 1,201,014 tests or 4389 tests per million of a 273 million population. It is one of the lowest testing rates in the world [7,8,9]. According to Allard and Lamb [10], a review of data, however, indicated that the number of deaths might be higher than the number reported as those who have died with acute symptoms but had not been confirmed to have COVID-19 were not yet counted in the official death figures. Since the first case was reported, the Indonesian government encouraged people who showed signs or had contracted COVID-19 to isolate themselves for 14 days. President Joko Widodo had urged Indonesians to work from home, learn from home, and worship at home to avoid the spread of COVID-19. Self-isolation in this context means staying at home to avoid contact with others. The president made this recommendation into a national policy determined through P.P. 21/2020 on Large-Scale Social Restrictions. This policy received much criticism and is considered a disaster because cases are still increasing [11]. 

The Semarang City Government has also implemented a self-isolation policy by establishing a Community Activity Restrictions (C.A.R.) policy starting 28 April 2020. The C.A.R. policy’s implementation was emphasizing the C.A.R. policy stated in Mayor Regulation No. 28 of 2020 concerning Guidelines for Implementing Restrictions on Community Activities. The restrictions on community activities (C.A.R.) have been implemented to handle the spread of COVID-19 in Semarang City. However, before enacting this regulation, self-isolation was only needed based on the mayor of Semarang’s appeal. This policy is to suppress the spread of COVID-19, which has not shown a graphical decline. Studies conducted by Spinelli and Pellino [12], Pinotti [13]; Laidlaw [14] and Mason [15], show that self-isolation could stop the transmission of COVID-19. It is a system of epidemiological surveillance for the cases of COVID-19 with no symptoms and potential COVID-19 disease carriers. 

Although self-isolation has proven effective, the implementation of a self-isolation policy should consider informal sector workers’ conditions, due to their low salary and low income. Of course, they will suffer from many difficulties. In this study, informal sector workers participate in the informal economy; they do not have access to secure work, benefits, welfare protection, or representation. They tend to be low-income workers, working as day laborers, and have no savings. These include tour leaders, online drivers, and shopkeepers who cannot work from home because they must work at their workplace. As Nicola et al. [16] also noted, self-distancing and self-isolation has reduced the workforce across all economic sectors, including primary, secondary, and tertiary sectors, and caused many job losses. The World Health Organization [17] said that the pandemic is now affecting 210 countries and territories. Vaccine discovery, appropriate treatment, and physical distance are ways to break the chain of transmission. Full or partial locking measures are applied throughout the world, affecting more than 5 billion people. For most of the two billion workers and business owners in the informal economy, stopping work or working remotely from home is not an option. Staying home means losing their jobs and livelihoods, i.e., “dying from hunger or the virus.” It is a real dilemma facing informal economy workers. 

In 2019, the number of workers in Indonesia working in the informal sector had reached 57 per cent or around 74 million workers. Out of that figure, as many as 25 million people, or about 9 per cent, live below the poverty line. The Purchasing Manager Index survey on the sub-component of labor in some sectors shows that the manufacturing industry is among the first to reduce work due to decreased production and factory closure due to low revenue figures [18]. Based on the online survey conducted part-time from 29 March to 13 April by Abdul Latif Jamel Poverty Action Lab [18] in early 2020, 55 per cent of male and female workers who had previously worked typically became unemployed because of COVID-19. Their income fell below the poverty line because the company where they used to work temporarily closed. If so, self-isolation affects marginalized vulnerable groups’ due to their income falling below the poverty line. The issues are how the vulnerable cope with these conditions, including how they think and act, and the solution from the government in anticipation of the situation so that the position of vulnerable groups does not worsen.

## 2. Literature Review

The debate over how to explain human behavior and examine the mind began in psychology. Structuralism and functionality were the first two psychological theories to emerge. In psychology, structuralism emphasizes on the breaking down of mental processes into their most fundamental components [19]. Functionalism is a psychological school of thought that arose from the Darwinian theory and focuses on the usefulness and purpose of behavior that has evolved over thousands of years of human life [20]. In line with the structuralism theory, this study uses the rational and non-rational model. Both concepts refers to the organization of the human mind, introduced by Lévi-Strauss [21] in his book, Structural Anthropology. This concept was discovered by Lévi-Strauss when examining the native tribe of Yanomamo who inhabit the South American rainforest region. They live in the Amazon rainforest between the border of Brazil and Venezuela. As their area of residence is very remote and unreachable, they were only recognized by outsiders at the beginning of the 20th century. The Yanomamo have now become one of the most studied ethnic groups in modern science. At Yanomamo, Lévi-Strauss found a unique organization of thought when they deal with illness. They heal diseases by magical means by making offerings to the spirit. They assume that the pain for them is due to an evil spirit disorder. It is called the healing method with a magic-religious model or non-rational model. They only knew how to cure medically (rational model) after the missionaries introduced modernization around the beginning of the 20th century.

Lévi-Strauss argued that in all human beings there is an essential genetic inheritance, i.e., structuring. It is the ability to structure, arrange a structure, or embed a particular form to the phenomena it faces. In everyday life, what is heard and witnessed by humans is an embodiment of this internal structure’s existence. However, this embodiment is never complete. A structure only manifests in a context, just as a sentence in language forms a language structure. The relations of language in the deep structure has simplified into binary oppositions (binary oppositions as organizations of thought and human culture, contribute to responding to reality). 

Many researchers developed the Lévi-Strauss model to study cultures such as [22]. Their studies focused on how structuralism is employed in exploring culture within a peasant society. Structuralism studies were applied to examine complex societies facing tragedies; in this case, the COVID-19 pandemic has not yet become much of a focus. Theoretically, when facing tragedies, humans still must live with reasoning and an adaptive structure of action so that they could stay alive. Therefore, this study seeks to use structuralism to find deep structures in mindset models reflected in vulnerable groups’ surface structures amid a pandemic. Based on the review of Lévi-Strauss’s [21] explanation on the structure of actions, how citizens respond to self-isolation policies is determined by their mindset, this becomes the cognitive structure of citizens (deep structures). Binary opposition’s perspective generally determines the way of thinking of citizens: non-rational or rational. 

For citizens controlled by rational thought, it is assumed that self-isolation is a sensible policy because the transmission of the virus is solely prevented by isolating oneself. At least, epidemiology has taught much information through the media. So, self-isolation is the recommended solution on a scientific basis to cut the transmission of the virus. People experience self-isolation due to the rational thinking models which guide them. Furthermore, the result of this method is compliance.

Meanwhile, residents who use non-rational thinking assume that avoiding COVID-19 does not mean having to isolate. They consider that the COVID-19 pandemic is God’s form of anger at human behavior, so, they decide not to self-isolate, but to worship God and apologize for their mistakes and sins. This action is influenced by a way of thinking called the magical religious model. It refers to a layperson’s perspective of a handicap as the result of one’s wrongdoings in this or previous lives, resulting in the wrath of supernatural powers and the penalties as the currently-acquired terrible condition [23]. The output of the model is that people do not have to be obedient and self-isolate. They are still doing routine activities to make money by asking for protection from God.

## 3. Materials and Methods

This qualitative study applies an ethnographic method. Ethnography is a type of research field that aims to learn about a specific setting or environment’s culture [24]. Ethnography frequently relies on participant observation over a lengthy period of time in the field, as well as other qualitative and quantitative methodologies [25]. For the goal of monitoring and documenting discussion and behavior, the researcher establishes ongoing interactions with research participants. In such circumstances, the researcher is the primary data gathering and analysis tool. The researcher wants to put specific occurrences in a larger, more meaningful context with a focus on the culture and social interaction of the people or groups being watched. Ethnography is especially useful for determining how social and cultural norms affect the success of health treatment [26]. It aims to explore and understand (verstehen) the reality and dynamics of views, beliefs, and strategies of the informants to overcome crises. The sample of this study is informal workers such as online and conventional drivers, shop assistants, and tour guides who cannot do work from home due to the nature of their workplace. Also, the pseudo-names are used for the informants. There are 25 respondents involved in this study. However, in-depth interviews were conducted with 4 informants working in the informal sectors. According to Creswell & Poth [24], in qualitative inquiries, 5 to 7 informants are sufficient for analyzing and drawing a conclusion. From 25 informants, 5 informants were tour guides, 10 informants were online drivers, 5 informants were conventional drivers, and 5 informants were shop assistants (refer to Table 1). This research was conducted from 15 March to 20 May 2020, with in-depth interviews collected by phone through the video call. The two-hour interviews consisted of questions and answers for each informant. The three main questions related to the cultural response of the informants toward COVID-19 and the self-isolation policy, i.e., Q1: The Informants Understanding or Interpretation (concerns the perspective, beliefs, and value system) toward COVID-19; Q2: The opinion of informants (related to their perspective and beliefs) on the implementation of a self-isolation policy by the Mayor; Q3: The attitude of informants toward the self-isolation policy (pleasing to the manifestation of belief in actual actions, such as accept vs. reject). Also, detailed demography profiles of informants, including gender, age, education, employment (informal sector), marital status, total income and informant’s vulnerability (income-related), were taken. The results of the interviews were recorded and then analyzed. The taxonomic analysis was applied to classify views and create patterns of economic vulnerability experienced by vulnerable groups. 

## 4. Results

### 4.1. Development Achievements of Semarang City

Based on data from the Bureau of Statistics Indonesia (B.P.S.) of Central Java Province, the achievement of macro development indicators in Semarang City in 2019 shows encouraging numbers. Many macro indicators can be seen including; (1) economic growth reaching 6.86 per cent; (2) a poverty rate of 3.98 per cent, while the average poverty rate in Central Java is 11.41 per cent; (3) an Open Unemployment Rate (T.P.T.) of 4.54 per cent while Central Java was 4.25%; and a Human Development Index (HDI) of 82.72 while the average in Central Java was 71.73. The HDI of Semarang City is far above the average of 35 regencies/cities in Central Java. High and low HDI numbers are measured from the components of Life Expectancy (UHH), Length of School Expectation (H.L.S.), Average Length of School (R.L.S.), and Purchasing Power Parity (P.P.P.).

Starting from some macro indicator achievements, compared to 35 other districts/cities in Central Java, Semarang City has a prominent achievement figure. However, during the crisis, this achievement was disrupted, especially regarding city inhabitants working in the informal sector such as tour guides, online and conventional drivers, and shop assistants. Based on B.P.S. figures, the Semarang City poverty line in 2019 is IDR. 474,930/month. The average income of those workers in the informal sector before the COVID-19 ranged from IDR. 1,000,000–IDR. 2,500,000. This amount of income was enough to meet their basic needs. However, it dropped extremely to IDR. 0–IDR. 1 million or below the poverty line due to the execution of Semarang city’s self-isolation policies. No income occurred due to the character of the work done that depends on the existence of consumers. This situation calls upon the study to explore and analyze their adaptation patterns to vulnerability, and their mindset and response to self-isolation policies executed.

### 4.2. Demography Profile of Respondent

This study uses informants, as many as 25 people who work daily in the informal sector. They have diverse backgrounds in terms of occupation, age, gender, marital status, and income. The variety of information about informants can be seen in Table 1.

Table 1 shows the informants’ classification according to the type of work, age, level of education, marital status, and monthly income. From Table 1, we found the four types of informants. First, five informants were tour leaders; they consisting of three informants aged 20–30 years old, two aged 40–50 years old. One was a high school graduate, and four informants did not complete their junior high school. Five informants are currently married with monthly incomes ranging from > IDR 0–IDR. 500,000. The second classification consists of ten informants of online motorcycle taxi drivers; six informants are aged 20–30 years old, four aged 31–40 years old and four graduated from junior high school, and six with elementary school education. Five informants are married, and six informants have an income with a range > 0 to five hundred thousand rupiahs monthly, and four informants earn > IDR. 500,000–IDR. 1,000,000/month. The third category is five informants of conventional motorcycle taxi drivers; four are aged 20–30 years old, one aged 31–40 years old, four graduating from senior high school, and six informants do not graduate from junior high school. In terms of marital status and income, five informants are married, with four earning >IDR. 0–IDR. 500,000/month and four informants earning IDR. 500,000–IDR. 1,000,000/month. The fourth classification is shop attendants totaling five informants consisting of four informants aged 20–30 years old, and one aged 31–40 years old; four graduated from Senior high School, and one did not get past junior high school. In terms of their marital status and income, five informants are married, with four informants earning IDR. 0–IDR. 500,000, and one earning IDR. 500,000–IDR. 1,000,000.

### 4.3. The COVID-19 Self-Isolation Policy towards Occupational among Vulnerable Groups 

#### Self-Isolation, Deep Structure, and Culture

This section reports the pattern of informal worker’s vulnerability due to COVID-19. As we known, COVID-19 hit all over the world and its given effect on tour guides and travel companies led to shops no longer operating. In addition, this study conducted in-depth interview with four informants, namely Uli, Hedi, Yudi and Narso. Question 1: The informants Understanding or Interpretation (concerns the perspective, beliefs, and value system) toward COVID-19. Uli answered, “*For me, COVID-19 is a dangerous type of virus. It attacks the respiratory tract which gives people a fever, high fever, and for a few weeks later, Covid can attack the lungs. The Covid is transmitted through sneezing, then transferred to other people and causes the person concerned exposing to Covid. This virus is categorized as very dangerous because it can cause death for those exposed to it. Thus, for the sake of the health of myself and others (children, husband, parents, neighbors, friends, among others), I need to obey a health protocol by wearing a mask, washing hands, keeping my distance from others.*”

Further, Hedi said, “*For me, Covid is a virus that must be avoided and dealt with by medical means. I got much information through the media on how to prevent it. So far, I have followed the government’s advice to cut the chain of this virus, so it doesn’t reach my family, namely by maintaining distance, wearing masks, and washing hands. In addition, pray, to pacify the mind.*”

Yudi said, “*Covid is a perilous virus, the risk of death. The disease must be avoided by obeying health procedure. Significantly if we are contaminated, things will be complicated. The cost of recovery is prohibitive, and this is not to mention that everyone close to us must also be isolated and checked. Actually, I was forced to work because my wife is only a housewife. If I don’t work, there’s no income. I feel insecure and afraid of the virus. That’s why, even though I am working, I still maintain the SOP for health protocol.*”

In addition, Narso answered, “*The virus is potent and changes all economic order and rules. Viruses are hazardous and can cause death for people who are contaminated. Diseases are like form and form; I have never seen a contaminated person, but I believe the condition exists. So, I should avoid it by maintaining Health procedures.*”

Furthermore, we asked also related informants’ opinion on the implementation of the self-isolation policy (Question 2). The first informant (Uli) answered, “*The transmission of the COVID-19 virus must be isolated so that it does not reach us. Various ways can be done, and I agree to undergo it according to medical procedures. Related to that, I often get information spread through WhatsApp Group, social media, radio-TV so that every citizen care about their health and others by obeying independent procedures. So, regarding the number of policies implemented by many people, I am delighted with the hope that the more people stay at home, the lower the potential for people to be positively affected by Covid.*”

Then, Hedi answered, “*…the self-isolation policy is indeed a bitter choice. According to him, if we are not following, it can harm us in terms of health. It’s not impossible; the virus transmitted through airborne sneezing caught us; while I didn’t recognize my passengers, whether they were immaculate of the virus or not. So, self-isolation is the best option.*”

Furthermore, Yudi said, “*This self-isolation makes people reluctant to leave the house; people choose to stay at home better. Even if someone leaves the house, they do the necessary activities. Even if shopping is only to meet daily needs. So, the place where I work was struck by two issues. First, due to the prohibition on Hajj and Umrah, second, due to the self-isolation policy. The shop is quiet. Store revenue is not what it used to be as before.*”

In addition, Narso answered, “*Implementing a self-isolation policy is not appropriate and can damage the economy of a lower class like me. There is no problem for groups who have permanent jobs, such as civil servants and private employees. But people who work like me are definitely in trouble. Mainly because of this rule people rarely go out and automatically I lost my customers.*”

Besides that, we also asked them Questions 3 which related to the attitude of Informants toward the self-isolation policy (Accept vs. Reject). Uli said, “*As a mother of a 16-year-old son, I feel I have to agree to this. Implementing a self-isolation policy is inappropriate and can damage the economy of a lower class like me. There is no problem for groups who have permanent jobs, such as civil servants and private employees. But people who work like me are definitely in trouble. Mainly because of this rule, people rarely go out and automatically; my customer were significantly reduced, I strongly agree, accept this policy. Imagine towards the affected person, as if life was on the verge of death. Even since a person self-isolates, since then, separation from family has occurred. The future seems bleak while the children are still small. Therefore, I prefer to follow government policies rather than dying in vain.*”

Then, Hedi answered, “*I have a family with one child aged two years. I still have parents (elderly) over 60 years old. I’m afraid they all caught the virus by us because of my ignorance. For that, I agree to follow self-isolation, accept it as a policy that helps others. In terms of income, I temporarily use the salary from my wife for shared expenses and occasionally deliver passengers by first making sure that the passengers obey the procedures when boarding the vehicle.*”

Furthermore, Yudi said, “*This isolation policy is good to minimize the possibility that people will be contaminated with the Covid virus. But, behind everything, the policy can drive the economy paralyzed. I don’t believe in and disobey the rules, but I also must support my wife and children. If you choose with common sense, it is a difficult choice. But I follow my heart that if I work for my family, my God will protect me. It means that now you surrender to God’s provisions. Whereas it is important to follow health procedures such as wearing masks, washing hands, and social distancing.*”

Lastly, Narso answered, “*Personally, I disagree with the self-isolation policy. It makes the difficulty to everyone. Because if I stay at home my family cannot survive, because I don’t earn any income. Conventional motorcycle taxi driver like mine is needed to find customers, not waiting for customers at home. The matter of life and death is fixed. The important thing is that we maintain health procedures, wear masks, wash hands and after work, shower and wash clothes. Just believe God will protect us, then pray and ask for his protection.*”

In the line with the result of interviews with four informants, this study found that in a condition of concern after the appeal for self-isolation, Uli complied with the government’s advice, isolating herself, staying at home, and not going to work. Even if she needs to go out, Uli will follow the government’s health protocol to keep a distance from others, wear a mask, wash hands, and take a bath on returning home. Complying with this protocol by Uli is immensely rational because she has a six-year-old child at home. She believes that COVID-19 is very dangerous for children and it has serious consequences and causes death. As a result of complying with this appeal, on the other hand, Uli lost her source of income. Uli has no income for three months. Previously she earned an average of IDR. 2,400,000 a month. The total income included in the category of the population who have income far above the poverty line (IDR. 474,930) in the city of Semarang.

Hedi, a 27-year-old man, married with one child, had a similar experience. He works as a car online driver. He said, “*I was forced to isolate myself, or at least take care of myself when carrying passengers because I was afraid of contracting the virus from the passengers.*” Initially, he was very strict in following the appeals of self-isolation, but he still must go to work because of his responsibility to meet his household needs. However, his income dropped sharply. Before the pandemic, he could earn more than IDR. 100,000 per day, with an average of 25 trips a day and a monthly income of IDR. 2,500,000. However, now his income is only IDR. 1,000,000 per month. It has decreased by around 60 per cent or IDR. 1,500,000 from his previous income. Therefore, Hedi experienced a situation where his income per month decreased dramatically due to his request for self-isolation.

Yudi is a 37-year-old man who has a family with one child. His wife is unemployed and a housewife. He worked as a shop assistant at a supply shop and souvenirs shop for Hajj and Umrah. Before COVID-19, his salary was IDR. 1,100,000 per month. However, due to the Umrah and Hajj pilgrimage travel restrictions, the shop could no longer afford to employ Yudi due to having no customers. The shop owner promised him that there would be a delay in payment of salaries while waiting for the conditions to return to normal. Yudi is an informant who has experienced income delays due to the crisis. Thus, the story experienced by each informant and their economic conditions is presented in Table 1 below. This table contains 25 informants, as interviewed, based on these professional classifications: five tour guides, ten online drivers, five conventional motorcycle taxi drivers, and five shop assistants.

Based on Table 2, the pandemic has a paradoxical effect on the household economy of vulnerable groups working in the informal sector. The pattern of vulnerability consists of three levels. First, those who experience a loss of income because their workplaces are no longer in operation. Second, those who experience a decline in revenue due to decreased purchasing power which impacts declining income. Third, those who experience delays in the payment of income because their workplace has experienced a decrease in income.

Table 2 shows that the conditions of vulnerability experienced by informal workers are diverse in terms of income. Diversity is related to the perspective that underlies the way they think and responds to policies of self-isolation. This fact is proved from the interviews with 25 informants by telephone (video call). Based on the taxonomic analysis, it was found that informants’ perspectives manifested into two categories. First, those who believed COVID-19 is a manifestation of God’s anger due to human behavior exploiting natural resources. To avoid contamination, they must go to pray and apologize for all their mistakes. If they apologize, then they will get protection from God from the dangers of COVID-19. Those who believe such methods are called adherents to the religious-magic model or the non-rational model. Most informants who have non-rational thinking patterns refuse to undergo isolation due to the requirement to meet the families’ needs. 

For this first type of response, the picture is as described by Narso (39 years old), an elementary school graduate, married with three children. Narso worked as a conventional motorcycle taxi driver operating at the Kagok motorcycle taxi station in Semarang. He believed the plague was God’s test for humans. Against this test, he felt terrified, and to keep himself safe he did ‘repentance’, prayed and carried out his obligations to God on time. For him, a virus is like a call to pray which allows him to understand the meaning of ‘going home’ to God. The second group are those who argue that the virus is a natural phenomenon, a type of disease originating from Wuhan. This virus spreads to other countries through the process of transmission from one person to another. Thus, the only way to avoid viruses is to self-isolate. They obey the obligation of self-isolation despite losing their income. They believe that this perspective will save them. Those who follow this perspective adhere to the rational model. When people ignore the self-isolation directive and do not believe that its application will save them, this is called the non-rational model. A complete response of the informants to self-isolation is seen in Table 3, below:

Table 3 shows the cultural responses of informal workers to the self-isolation policy, the variation of perspectives, namely rational and non-rational in each type of informant, and profession. Nevertheless, the number of informants who have non-rational ideas is more dominant compared to those with rational views. Generally, an informant with a non-rational perspective comes from a low educational background and they tend to ignore self-isolation. Meanwhile, those who have a rational view are generally more highly educated and this informant manages to comply with self-isolation provisions. Thus, the appeal for self-isolation has given rise to binary opposition from informant actions and ways of thinking. In line with the findings, most of informants were categorized as non-rational thinking due to their education level. However, the government can solve the issue with the non-rational thinkers by offering assistance. Assistance that focuses on daily goods, such as rice, eggs, fried oil etc., will encourage people to stay at home instead of going out to work. Besides the non-financial assistance, the government needs to increase their attention on who is breaking the rules and take decisive action against them to stop COVID-19 transmissions. Self-isolation has also brought down most of the business sector, especially the informal sector. Many studies show outbreaks experienced by a country lead to the informal sector’s contraction [27,28,29].

## 5. Discussion

### Self Isolation, Deep Structure, and Cultural Response

Self-isolation is the sine qua non, whether it is carried out because of the government’s policy or independently by a person. Self-isolation is a control system that emerged to prevent people’s movement over a particular period to avoid disease transmission [30,31,32]. It is identical to the self-quarantine of a person or group of people due to the pandemic. In epidemiological discourse, the term self-isolation is often equated with medical quarantine where individuals that have been infected are isolated so that they do not transmit disease to others. Although the concept of quarantine was created to prevent the spread of animal diseases, quarantine is also used to avoid the spread of disease among humans [33]. However, based on field observation, self-isolation is an effort to avoid transmission that affects vulnerable groups’ income. In this case, vulnerable groups earn an average above the poverty line, rely on daily facts, do not have savings, and are in debt. It is an inherent part of vulnerable groups’ lives, and they mostly work in the informal sector [18].

Notable impacts experienced by the vulnerable groups categorizes the groups into three vulnerable sub-groups. First, those who experience job and income lost. This is experienced by informants who work as tour guides. Their work is very much dependent on tourists; therefore, the pandemic causes the tourism sector to no longer be able to operate. Second, the group experienced a decrease in income due to the decline in their purchasing power because of a declining income. This category involves informants who work both as online and conventional motorcycle taxis. Their work is highly dependent on consumers who use motorcycle taxi services. However, COVID-19 has caused most residents to choose to stay at home. That is why motorcycle taxis drivers experience a decrease in income of between 50–60 per cent. Third, those who experience delayed payment of wages due to workplaces receive decreased income. This category is experienced by informants who work as shop assistants whose business types are also affected by COVID-19, such as stores that provide hajj equipment. During the pandemic, the activities of Hajj and Umrah has stopped temporarily. This causes the store’s income to drop dramatically. Therefore, the shopkeeper chooses to lay off employees and delay salary payments until the conditions better.

To respond to the policy of self-isolation, each vulnerable group has a specific variation in its response. Some reject self-isolation, which means ignoring the obligation to stay at home. Meanwhile, many received self-isolation accompanied by disciplinary action following the obligation to stay at home until stipulatory time. Diversity of responses is a consequence of circumstances that indicate an order in the human mind’s organization. For Lévi-Strauss [21], this is not impossible due to a primary human ability that is inherited genetically. This ability exists in all humans, namely the ability to structure or construct a structure on the phenomena encountered.

Furthermore, the structure in the form of an action pattern is a surface structure that reflects the human’s deep structure of a human mind. The internal structure contains relations of ideas, concepts, and thoughts or a cultural phenomenon that is related to several other phenomena that, at a certain point, determine the meaning of the phenomenon. The relationships that exist in the internal structure can be simplified into a binary opposition model. It is relevant to Lévi-Strauss’s thought (1976) that cultural studies need to be directed at how the human mind’s mechanism works and reflects on the structure. The human mind’s mechanism works and reflects on the surface structure. According to Lévi-Strauss [21], the study of natural thought processes has been heavily influenced by unnatural conditions. The study of fairy tales (myths) by Sturrock [34] is often put forward because it embodies a simple community mind where little things are found. The similarity is the foundation of Lévi-Strauss’s study of human reasoning. Another reason for studying myths is that they are similar to languages where myths and languages are both communication media to convey messages, language and aspects of myths demonstrated by the presence of myths in reversible and non-reversible time. It also proposed a view because the meaning in language lies in the combination of phonemes. Myth is examined by looking at the combination of various characters and their actions and their respective positions in the combination. This equation of myth and language led Lévi-Strauss to issue a theory that said, “myth is language, functioning on an especially high level where meaning succeeds practically at ‘taking off’ from the linguistic ground on which it keeps on rolling.”

For Lévi-Strauss [21], the study of culture is not merely revealing the surface structure as a systemic reflection of the workings of the human mind’s organization but at the same time as a framework for cultural studies is carried out through the disclosure of two types of structures, namely deep structures and surface structures simultaneously. In this study, the inner structure is known through tracing discourse, which shows the mindset as characterized by binary opposition. The concept contains ideas and ideas in the formula of magic-religion models (non-rational models) and rational models.

Based on field observations, two types of reasons have been found with binary opposition in different vulnerable groups. On the fieldwork basis, it is found that the way they responded to self-isolation; a resistance to self-isolation, ignoring the obligation to carry out health protocols, and ignored to stay at home. For them, fulfilling basic needs is jihad fisabilillah, while life and death are a matter of God. Reasoning thought has become a deep structure for vulnerable loss income groups. However, not all of these vulnerable groups are irrational. Some have rational reasons to believe that safety and health are more important than income. Health for them is an investment in life, while income is only something for a living. This mindset is found in vulnerable income groups with a background in Junior High School education. Those who have consciousness, undergo adaptive isolation themselves with a new way of life and this perspective becomes a deep structure among those on loss incomes manifested in accepting self-isolation policies. 

Vulnerable groups in decline income experience have the same expression for informants working as an online motorcycle taxi and conventional motorcycle taxi. This group is also divided into two sub-logics, namely the magic-religion model and the rational model. For those with a non-rational model of reasoning, their educational background is in elementary school. They ignore the obligation to isolate themselves, as well as health protocols. Looking for a living is the main thing based on the intention to do jihad fisabilillah. It believed that they would avoid the threat of COVID-19 and other threats due to a belief in God. The vulnerable group has lost the income among those who have rational thought. Like those who reason, they are educated to graduate from junior high school, carry out self-isolation, and follow the health protocol. These reasoning thoughts have become a deep structure among the decline-income group. The same pattern also manifests in vulnerable groups in the category of delay-income for informants who work as shop assistants. 

The response pattern is the same as the other two groups above, those who have the magic-religion mind and rational model. Those who adhere to non-rational reasoning models generally also have an elementary education background. The tendency is also to neglect self-isolation and disregard the health protocols. The Daqlam things of earning a living also share the view of the two groups mentioned above—some in the group have the same mindset, where this logical reasoning is the deep structure. Self-isolation has thus been significant to a new cultural structure among the vulnerable workers in the informal sector. It is a consequence of the implementation of self-isolation policies that tore up most of the informal sector. Much research states that citizens’ isolation from outbreaks generally leads to a contraction in the informal sector [35,36,37].

Many solutions propose to overcome this problem. For example, Israr et al. [37] suggest handling the vulnerable groups when facing outbreaks in two ways, such as meeting basic needs and providing facilitation to them to have sustainable livelihoods to independently re-income. Relevant to the implementation of self-isolation for a vulnerable group, the government is expected to provide a policy breakthrough to provide facilitation support suggested by Israr et al. [31]. It is done by providing social security assistance programs by providing direct cash to overcome difficulties in meeting basic needs from the government [28,29]. It focused on distributing the budget and implementation of poverty alleviation programs and encouraging the realization of health insurance covering all residents, particularly low-income families. The insurance system can guarantee all levels of society regardless of economic vulnerability and give them good quality health services and social protection. To date, the health insurance borne by the Social Security Organizing Agency (BPJS) has not completed everything because it only serves the public who pay monthly contributions. Community groups who work in the informal sector with low income are not included in receiving contributions. Delay in paying dues can have an impact on termination and providing health services to them. 

The government also needs to allocate public funds to strengthen basic health services for the poor. These services include access to health education, clean water, nutrition, immunization and various infectious and non-communicable diseases, it is a social security protection program provided to vulnerable groups to have income or sustainable livelihoods. Other examples of programs are the expansion of social assistance, pre-employment cards, the formation of BUMDES (Village-Owned Enterprises). Besides assisting in the cost of electricity and free water and tax breaks for workers and MSMEs. An economic recovery program for the business world is also related to the informal sector [37]. Without adequate support from the government, vulnerable groups and low-income informal workers will experience increasingly severe vulnerabilities and this could lead to the birth of an unfavorable situation. While, on the other hand, they also belong to vulnerable groups at risk of contracting COVID-19 and can potentially spread the infection further.

## 6. Conclusions

The current COVID-19 pandemic, which continues to spread, has a paradoxical effect on informal sector workers. Those who are members of vulnerable groups experience a decline, delay, and loss of income due to the implementation of the self-isolation policy. Thus, the self-isolation policy has divided the informants’ mindsets into rational and non-rational thinking models. The mindset model is related to the informant’s educational background. The rational mindset applies to informants who stick to appeals for self-isolation. Non-rational ones are related to informants who are forced to violate appeals due to family responsibilities, even though their income is still insufficient or below the Semarang poverty line. The finding of this study has two implications, namely practical and theoretical implication. Practically, the government and community (vulnerable groups) need to choose the right strategy to address this issue and reduce the impact of COVID-19 through the implementation of a self-isolation policy which is more precisely and on target. In this context, the researchers mention two strategies that need to be carried out together. First, strategies to access basic needs with a focus on direct assistance to target vulnerable groups. Second, provide assistance that can facilitate vulnerable groups to support their sustainable livelihoods. 

Theoretically, this research was apparent from the review of the literature that the impact of the COVID-19 self-isolation policy on the occupations of vulnerable groups by considering a rational or non-rational thinking model was still unclear and limited. Therefore, this study contributes to the literature by finding evidence to support the theoretical foundation of the impact the COVID-19 self-isolation policy on the occupations of vulnerable groups by considering a rational or non-rational thinking model. Also, filling the gap in the knowledge that was identified in previous studies [4,6,7,8,9,16,17,31,32,38,39] regarding impact of the COVID-19 self-isolation policy on the occupations of vulnerable groups by revealing that self-isolation, deep structure and cultural responses do have a relationship with rational or non-rational thinking models, whereas the individual would be making decisions on the basis of the human minds [21,22,34]. In addition, this study contributes to the theory, whereas the findings of this study are confirmed that the individual’s decision depends on rational or non-rational thinking (Human Minds) and cultural background. Furthermore, this research discusses how the government formulates policies for vulnerable groups due to COVID-19 and its impact on their occupation. Thus, the government needs to develop anthropology policy reforms. Of these, this study only discusses how to formulate “public policies” that have been agreed upon in advance, to protect the layers of society (i.e., vulnerable groups) to improve their quality of life, including health through self-isolation policy implementation. Also, allowing them to stay healthy and productive during the COVID-19 pandemic.

## Figures and Tables

**Table 1:** Profile of Informants.

Type of Profession	Age		Education		Marital Status		Income	
	a	b	a	b	a	b	a	b
Tour Guide	3	2	4	1	5	0	3	2
Online Driver	6	4	6	5	5	0	6	4
Conventional Driver	4	1	4	1	5	0	4	1
Shop Assistant	1	4	4	1	5	0	3	2

Note: Age: a. 20–30, b. >30–40, Education a. <S.D., b. S.M.P. and above (S.M.A.), Marital Status; a. Married. b. Unmarried, Income/month: a. IDR 0–IDR 500,000, b. > IDR500,000–IDR 1,000,000.

**Table 2:** The informant’s income before and during the COVID-19 pandemic, and income status.

Type of Profession	Frequency	Income before the Crisis (IDR)	Income during a Crisis (IDR)	Income Status
Tour Guide	5	2,400,000	-	No Revenue
Online Driver	10	2,500,000	1,000,000	Decreased Revenue
Conventional Driver	5	1,500,000	750,000	Decreased Revenue
Shop Assistant	5	1,100,000	-	Wage Payments Postponed

**Table 3:** The cultural responses of informal workers to the self-isolation policy.

Type of Profession	Vulnerable Category	Education	Frequency	Perspective	Cultural Response to Self-Isolation
Tour Guide	Lost Income	<JHS	4	Non-Rational	Ignore
		>JHS	1	Rational	Obey
Online Driver	Lost Income	<JHS	6	Non-rational	Ignore
		>JHS	5	Rational	Obey
Conventional Driver	Decline in Income	<JHS	4	Non-rational	Ignore
		>JHS	1	Rational	Obey
Shop Assistant	Delay Income	<JHS	4	Non-rational	Ignore
		>JHS	1	Rational	Obey

Note: JHS is Junior High School.

## References

[1] Economics in the Time of COVID-19: A New eBook. https://voxeu.org/article/economics-time-covid-19-new-ebook.

[2] WHO (2020). Situation Report-82 Highlights. https://www.who.int/docs/default-source/coronaviruse/situation-reports/20200411-sitrep-82-covid-19.pdf.

[3] Yan C.H., Faraji F., Prajapati D.P., Boone C.E., DeConde A.S. (2020). Association of chemosensory dysfunction and COVID-19 in patients presenting with influenza-like symptoms. Int. Forum Allergy Rhinol..

[4] Zhou S., Zhang L.-G., Wang L.-L., Guo Z.-C., Wang J.-Q., Chen J.-C., Liu M., Chen X., Chen J.-X. (2020). Prevalence and socio-demographic correlates of psychological health problems in Chinese adolescents during the outbreak of COVID-19. Eur. Child Adolesc. Psychiatry.

[5] Wang Q., Su M. (2020). A preliminary assessment of the impact of COVID-19 on environment—A case study of China. Sci. Total Environ..

[6] Djalante R., Lassa J., Setiamarga D., Sudjatma A., Indrawan M., Haryanto B., Mahfud C., Sinapoy M.S., Djalante S., Rafliana I. (2020). Review and analysis of current responses to COVID-19 in Indonesia: Period of January to March 2020. Prog. Disaster Sci..

[7] Ting D.S.W., Carin L., Dzau V., Wong T.Y. (2020). Digital technology and COVID-19. Nat. Med..

[8] Whitelaw S., Mamas M.A.A., Topol E., Van Spall H.G. (2020). Applications of digital technology in COVID-19 pandemic planning and response. Lancet Digit. Health.

[9] Pan X.B. (2020). Application of personal-oriented digital technology in preventing transmission of COVID-19, China. Ir. J. Med. Sci..

[10] Allard T., Lamb K. (2020). Exclusive: More Than 2200 Indonesians Have Died. Google Scholar. https://scholar.google.com/scholar?hl=en&as_sdt=0%2C5&q=Exclusive%3A+More+than+2%2C200+Indonesians+Have+Died+with+Coronavirus+Symptoms%2C+data+shows%2C++https%3A%2F%2Fwww.reuters.com.&btnG=.

[11] Bean J.P. (2020). Indonesia’s ‘New Normal’: A Disaster in the Making. Asia Times.

[12] Spinelli A., Pellino G. (2020). COVID-19 pandemic: Perspectives on an unfolding crisis. Br. J. Surg..

[13] Pinotti F., Di Domenico L., Ortega E., Mancastroppa M., Pullano G., Valdano E., Boelle P.-Y., Poletto C., Colizza V. (2020). Lessons learnt from 288 COVID-19 international cases: Importations over time, effect of interventions, underdetection of imported cases. medRxiv.

[14] Laidlaw T. (2019). Pandemic Stories: Rhetorical Motifs in Journalists’ Coverage of Biomedical Risk. Minerva.

[15] Mason K.A. (2015). H1N1 Is Not a Chinese Virus: The Racialization of People and Viruses in Post-SARS China. Stud. Comp. Int. Dev..

[16] Nicola M., Alsafi Z., Sohrabi C., Kerwan A., Al-Jabir A., Iosifidis C., Agha M., Agha R. (2020). The socio-economic implications of the coronavirus pandemic (COVID-19): A review. Int. J. Surg..

[17] World Health Organization COVID-19 Health Equity Impact Policy Brief: Informal Workers. http://www.who.int/about/licensing.

[18] Ramadhana M.R. (2020). A dataset for emotional reactions and family resilience during COVID-19 isolation period among Indonesian families. Data Brief.

[19] Blumenthal A.L., Danziger K. (2001). Wilhelm Wundt in History: The Making of a Scientific Psychology.

[20] Hergenhahn B.R., Henley T. (2013). An Introduction to the History of Psychology.

[21] Levi-Strauss C. (1976). Structural Anthropology.

[22] González-Ruibal A. (2006). House societies vs. kinship-based societies: An archaeological case from Iron Age Europe. J. Anthropol. Archaeol..

[23] Winkelman M.J. (1990). Shamans and Other “Magico-Religious” Healers: A Cross-Cultural Study of Their Origins, Nature, and Social Transformations. Ethos.

[24] Lewis I.M. (2004). Social and Cultural Anthropology in Perspective.

[25] Aktinson P., Hammersley M. (1998). Ethnography and participant observation. Strategies of Qualitative Inquiry.

[26] Schensul S.L., Schensul J.J., LeCompte M.D. (1999). Essential Ethnographic Methods: Observations, Interviews, and Questionnaires.

[27] Aji R.H.S., Subekti R.D., Nurhayati T. Indonesian Women: Emancipation Evidence against Global Pandemic. www.covid19.go.id.

[28] Amirudin A. (2019). Environmental Issues in Journalism Coverage at the Suara Merdeka Newspaper. The 4th International Conference on Energy, Environment, Epidemiology and Information System (ICENIS 2019), Tembalang, Semarang, Indonesia. E3S Web Conf..

[29] Hidayattuloh M.H., Bambang A.N., Amirudin A. (2019). Environmental-based budget planning in The Tegal Regency Government. The 4th International Conference on Energy, Environment, Epidemiology and Information System (ICENIS 2019), Tembalang, Semarang, Indonesia. E3S Web Conf..

[30] Anser M.K., Yousaf Z., Khan M.A., Nassani A.A., Alotaibi S.M., Abro M.M.Q., Vo X.V., Zaman K. (2020). Does communicable diseases (including COVID-19) may increase global poverty risk? A cloud on the horizon. Environ. Res..

[31] Bodrud-Doza M., Shammi M., Bahlman L., Islam A.R.M.T., Rahman M. (2020). Psychosocial and Socio-Economic Crisis in Bangladesh Due to COVID-19 Pandemic: A Perception-Based Assessment. Front. Public Health.

[32] Shammi M., Bodrud-Doza M., Islam A.R.M.T., Rahman M. (2020). COVID-19 pandemic, socioeconomic crisis and human stress in resource-limited settings: A case from Bangladesh. Heliyon.

[33] Quach H.-L., Hoang N.A. (2020). COVID-19 in Vietnam: A lesson of pre-preparation. J. Clin. Virol..

[34] Sturrock J. Structuralism and Since: From Levi Strauss to Derrida. https://pdfs.semanticscholar.org/f616/23a80ced41b5ad358e6855d00e1a487d2f47.pdf.

[35] Lui R.N., Wong S.H., Sánchez-Luna S.A., Pellino G., Bollipo S., Wong M., Chiu P.W.Y., Sung J.J.Y. (2020). Overview of guidance for endoscopy during the coronavirus disease 2019 pandemic. J. Gastroenterol. Hepatol..

[36] Kartika F.D.S., Helmi M. (2019). Meta-analysis of community’s adaptation pattern with tidal flood in Pekalongan City, Central Java, Indonesia. The 4th International Conference on Energy, Environment, Epidemiology and Information System (ICENIS 2019), Tembalang, Semarang, Indonesia. E3S Web Conf..

[37] Israr M., Khan H., Jan D., Ahmad N. (2014). Livelihood Diversification: A Strategy for Rural Income Enhancement. J. Financ. Econ..

[38] Syaifullah J., Syaifudin M., Sukendar M.U., Junaedi J. (2021). Social Media Marketing and Business Performance of MSMEs During the COVID-19 Pandemic. J. Asian Financ. Econ. Bus..

[39] Hantoko D., Li X., Pariatamby A., Yoshikawa K., Horttanainen M., Yan M. (2021). Challenges and practices on waste management and disposal during COVID-19 pandemic. J. Environ. Manag..

